# Novel magnetic nanosorbent graphene oxide-based for the automatic and simultaneous determination of arsenic and antimony by MSPE-FI-HG-ICP-MS

**DOI:** 10.1007/s00604-026-08161-w

**Published:** 2026-06-04

**Authors:** Irene Morales-Benítez, Álvaro Doblado-Onieva, Irene Sánchez-Trujillo, Carlos Vereda-Alonso, M. Mar López Guerrero, Elisa I. Vereda Alonso

**Affiliations:** 1 Department of Analytical Chemistry, Faculty of Sciences, University of Málaga, Campus de Teatinos, Málaga, 29071 Spain; 2 Instituto Universitario de Materiales y Nanotecnología, IMANA, University of Málaga, Campus de Teatinos, Málaga, 29071 Spain; 3 Department of Chemical Engineering, Faculty of Sciences, University of Málaga, Campus de Teatinos, Málaga, 29071 Spain

**Keywords:** Magnetic solid phase extraction, Nanosorbent, Graphene Oxide, Arsenic, Antimony, Hydride Generation, ICP-MS

## Abstract

Antimony and arsenic are two pollutants known for their bioaccumulative effects and toxic properties even in low concentrations, therefore there is a need to develop efficient and reliable extraction and preconcentration methods of these analytes in water systems simultaneously. In this work, an online ultratrace extraction and preconcentration methodology for environmental waters was carried out by combining magnetic solid phase extraction (MSPE), hydride generation (HG) and inductively coupled plasma mass spectrometry (ICP-MS). A novel nanosorbent, called M@GONIO, based on graphene oxide and magnetic nanoparticles functionalized with ammonium groups was designed, synthesized, and characterized for MSPE. The developed method was carefully optimized through univariate analysis and response surface methodology based on a central composite design. Under the optimized conditions, the enrichment factors for As and Sb were 84.7 and 84.6, respectively. The intra-day and inter-day precision expressed as relative standard deviation (RSD) were below 5.1% in all the evaluated conditions. The proposed analytical method was successfully validated and applied to the determination of trace of As and Sb in environmental waters from diverse origins.

**Graphical abstract:**

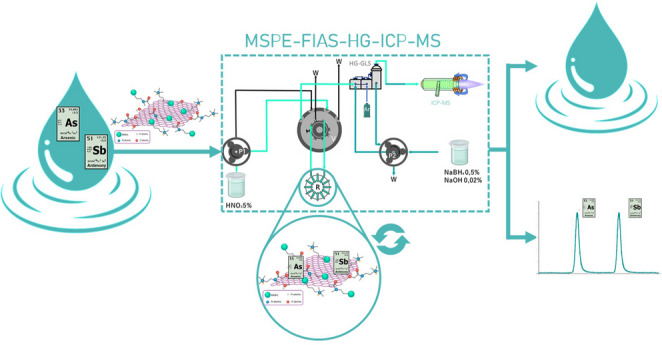

## Introduction

Environmental pollution in water is one of the main concerns of our society as it is reflected in the European Sustainable Development Goals (SDG) [1]. Contaminants of emerging concern have become a major focus since their presence in waters may have adverse effects on human health and aquatic life. The widespread occurrence and severe toxicity of arsenic (As) and antimony (Sb) in aquatic systems have become issues of considerable concern for environmental and public health authorities worldwide. Both elements are classified as priority pollutants by the U.S. Environmental Protection Agency (EPA) due to their carcinogenic potential, mobility in aquatic environments, and ability to bioaccumulate through food chains [2, 3]. In natural and industrially-influenced waters, arsenic and antimony typically exist in two main oxidation states: As(III)/As(V) and Sb(III)/Sb(V). Among these, the trivalent forms, arsenite and antimonite, are significantly more toxic and mobile than their pentavalent counterparts [4]. Despite these differences, EPA regulations only determine limits for total As and total Sb, established in 10 and 6 µg L^−1 ^respectively. Anthropogenic inputs from mining, smelting, and industrial processes have exacerbated the contamination of surface and groundwater with these toxic elements [5]. Monitoring and remediation efforts require not only the quantification of total As and Sb but also accurate speciation analysis to assess the environmental impact and implement suitable treatment strategies. However, the determination of trace levels of As and Sb in complex environmental matrices remains a challenge due to their low concentrations, diverse chemical forms, and potential interferences from coexisting ions. There are several detection techniques such as hydride generation atomic absorption spectrometry (HG-AAS), graphite furnace AAS (GFAAS), and inductively coupled plasma mass spectrometry (ICP-MS), which offer high sensitivity but often require preconcentration and matrix separation steps to reduce interferences and improve detection limits [6, 7]. In this context, magnetic solid-phase extraction (MSPE) has emerged as a powerful technique combining high selectivity, rapid phase separation, and environmental friendliness [7–9]. In most reported applications, MSPE is performed in an off-line configuration prior to instrumental detection (e.g., AAS, ICP-OES, ICP-MS), which enables analyte preconcentration and matrix simplification. Conventional off-line systems often involve multiple manual steps, including sorbent dispersion, separation, elution, and transfer, which not only increase analysis time but also introduce potential sources of analyte loss, contamination, and variability [10].

Magnetic nanomaterials, especially Fe₃O₄ nanoparticles, are widely used as sorbents due to their superparamagnetic behaviour, low toxicity, and ease of surface modification [11–14]. However, unmodified magnetite exhibits poor selectivity and stability, prompting the development of hybrid and functionalized composites to improve performance. Recent advances include the integration of Fe₃O₄ with carbon-based nanomaterials like graphene oxide (GO) [15], multi-walled carbon nanotubes (MWCNTs) [16], metal oxides such as MnO₂, Al₂O₃, and Au [17], or metal organic frameworks [10]. These composites exhibit improved dispersibility, greater surface area, and enhanced metal adsorption capacity.

The functionalization of magnetic nanoadsorbents with ligands such as amino, thiol, carboxyl, and imprinted polymers has proven to be an effective strategy for enhancing selectivity toward specific oxidation states of As and Sb [18]. For example, magnetic ion-imprinted polymers (MIIPs) have shown high affinity for Sb(III) due to the template-specific interaction during polymer synthesis [19]. Similarly, thiol-functionalized GO-based composites have been developed for selective As(III) adsorption, whereas amine-functionalized surfaces favor As(V) binding [20].

The anionic nature of arsenic and antimony species in aqueous media poses a significant challenge for their efficient adsorption using conventional nanoparticles [21]. In this work, a novel magnetic graphene oxide nanocomposite functionalized with quaternary ammonium groups (named M@GONIO) has been successfully synthesized and thoroughly characterized, this nanomaterial was specifically engineered to enhance interaction with these oxyanions. The incorporation of cationic functional groups provided enhanced affinity and dual selectivity toward both As and Sb species, ensuring efficient capture under environmentally relevant conditions. Beyond its intrinsic sorption capacity, the nanomaterial displayed excellent stability, dispersibility, and magnetic responsiveness, which are key features for its integration into automated MSPE platforms. Although several methods have been reported for the individual determination and speciation of As or Sb, the simultaneous extraction and quantification of both elements using an automated single MSPE platform remains largely underexplored.

In this work, we present a novel and optimized MSPE protocol coupled online to HG-ICP-MS for the simultaneous extraction and determination of inorganic arsenic and antimony species in natural and wastewater samples. By employing a new hybrid magnetic nanocomposite with tailored surface functionality (M@GONIO), the developed method offers high selectivity, sensitivity, and environmental compatibility. Analytical parameters were systematically optimized, and the method was validated with spiked and certified samples, demonstrating excellent accuracy, reusability, and matrix tolerance. The proposed method was successfully applied to the determination of trace of As and Sb in environmental waters: sea water, river water, wastewater treatment plant (WWTP), spring water and tap water. Our approach bridges a significant methodological gap and offers a valuable tool for routine environmental water monitoring of metalloid contaminants.

## Experimental

### Instrumentation

Measurements were performed using a quadrupole-based inductively coupled plasma mass spectrometer, ICP-MS NexION 2000 (PerkinElmer, Waltham, MA, USA), equipped with an AS-91 Autosampler (PerkinElmer, Waltham, MA, USA). The system was directly coupled to a FIAS 400 AS system (PerkinElmer, Waltham, MA, USA) which includes two peristaltic pumps, a five-port rotary valve, and a hydride generation-gas-liquid separator (HG-GLS, PerkinElmer, Waltham, MA, USA). All components were controlled by Syngistix software. Instrumental operating conditions and analytical parameters are summarized in Table 1. Daily performance checks were conducted, and the instrumental parameters were adjusted so that Ce^++^ (70)/Ce^+^ (140) and CeO^+^ (156)/Ce^+^ (140) ratios were equal to or less than 0.03 and 0.025, respectively.

**Table 1:** ICP-MS operation conditions

ICP-MS Instrument	NexION-2000 (PerkinElmer)
Monitored signals	^75^As, ^121^Sb
RF power, W	1600
Nebulizer Gas flow, L min^− 1^	0.9
Plasma Gas flow, L min^− 1^	15
Auxiliary gas flow, L min^− 1^	1.2
Sample introduction system	Perkin Elmer Gas-Liquid Separator (GLS)
Sampler cone	Ni
Skimmer cone	Ni
Torch alignment, mm (horizontal, vertical, depth)	-0.07/0.84/0.00

The HG-GLS was used with a 0.45 μm PTFE Fluoropore™ membrane filter (Merck Milipore, Tullagreen, Ireland) and enabled the conversion of ionic As and Sb into their volatile hydride forms to be introduced directly to the ICP-MS torch.

For on-line MSPE, a reactor containing 50 mg of M@GONIO packed into a PTFE tube (500 mm × 0.5 mm i.d.) knotted around a Nd/Fe/B toroidal magnet (40 mm o.d. x 23 mm i.d.t x 5 mm height; holding strength: 81.4 N) and positioned between two identical toroidal magnets (Supermagnete, Gottmadingen, Germany). Two polyethylene frits (Omnifit, Cambridge, UK) were placed at both ends of the PTFE tubes to prevent material loss. This reactor was installed in the sample loop of the five-port rotary valve of the FIAS 400 system. Samples, reagents, and wastes were delivered using PTFE pump tubing.

For M@GONIO characterization, infrared (IR) spectra were recorded using a Spectrum 100 FTIR spectrometer (PerkinElmer, Waltham, MA, USA). Samples were measured as potassium bromide (Merck, Darmstadt, Germany) pellets containing around 1% (wt/wt) of the material. X-Ray photoelectron spectroscopy (XPS) analysis was performed using a PHI 5700 instrument (Physical Electronics, Eden Prairie, MN, USA) equipped with a Mg Kα X-ray excitation source (hν = 1253.6 eV) was employed; binding energies (BE) were referenced respecting to the position of the C 1 s peak at 284.8 eV. The residual pressure in the analysis chamber was maintained below 3·10^− 9^ Torr during data acquisition. The microstructure was characterized by TEM using a JEM-1400 transmission electron microscopy (JEOL, Tokyo, Japan), SEM using a JSM-6490LV (JEOL, Tokyo, Japan) and N_2_ adsorption isotherms registered with ASAP 2020 V4.02 (Micromeritics, Norcross, GA, USA); equilibration interval (EI) of 20 s. The composition of the material (C, N, O, S) was determined by CHNOS elemental analysis from TruSpec Micro CHNSO (LECO, St. Joseph, MI, USA). For mass spectrometry analysis, GC-MS Trace GC Ultra / ITQ 1100 system (Thermo Scientific, Waltham, MA, USA) was employed using a retention time (RT) of 0.93 min and normalized intensity (NL) of 1.06E7).

### Reagents, standards and samples

All experiments were carried out with reagents and standards of analytical grade or superior. Doubly de-ionized water (DDW, 18 MΩ cm) obtained from a Milli-Q water system (Millipore, Bedford, MA, USA) was used throughout the study. All plastic and glassware were cleaned with nitric acid, stored immersed in 10% (v/v) nitric acid (Merck, Darmstadt, Germany); and rinsed several times with DDW immediately before use.

Calibration standards were prepared from 1000 mg L^− 1^ stock standard solutions of As^III^ (Merck, Darmstadt, Germany) and Sb^V^, prepared from potassium hexahydroxoantimonate (V) (Merck, Darmstadt, Germany). Standards of working strength were freshly prepared immediately prior to use by appropriate dilution. For pH optimization study, solutions with pH ≤ 2 were adjusted with diluted hydrochloric acid; pH values from 3.0 to 6.0 were adjusted using glycine–HCl and CH_3_COOH/CH_3_COONa buffers; from 7.0 to 10.0 employing H_2_PO_4_^−^/HPO_4_^2−^ and NaOH/HPO_4_^2−^ buffers; and pH ≥ 11 was achieved with NaOH solution. All these reagents were purchased from Merck (Darmstad, Germany).

To achieve As and Sb hydrides, a solution of 0.5% (wt/wt) NaBH_4_ (Acros Organic, Geel, Belgium) and 0.1% (wt/wt) NaOH was employed, the elution of the metal species from the reactor was achieved with a 5.5% (wt/wt) HNO_3_ solution.

For the synthesis of M@GONIO, ferric chloride hexahydrate (FeCl_3_·6H_2_O), ferrous chloride tetrahydrate (FeCl_2_·4H_2_O), ammonia 30% (wt/wt), methanol, sodium chloride, iodomethane, acetonitrile, glacial acetic acid and H_2_SO_4_ 98% (Merck, Darmstadt, Germany) and H_2_O_2_ 35% (Scharlab, Barcelona, Spain) were used. 3-Aminopropyltriethoxysilane (Fluka, Buchs, Switzerland). Tetraethoxysilane (TEOS), N,N-dicyclohexylcarbodiimide (DCC), glutaraldehyde, ethylenediamine (EDA), graphite, NaNO_3_ and KMnO_4_ (Aldrich Chemie, Steinheim, Germany), and ethanol (Carlo Erba, Milano, Italy). were also employed.

The certified reference materials (CRMs) analyzed to check the accuracy of the proposed procedure were TMDA 64.3 Fortified Lake Water (National Research Council of Canada, Ontario, Canada) and SPS-WW2 Batch 112 (Spectrapure Standards, Oslo, Norway).

#### Sample preparation

The applicability of the method was evaluated using several water samples from diverse sources including seawater (Plomo Cove, Almería, Spain), river water (Tinto River, Huelva, Spain; Alcaucín, Spain and Ill River, Strasbourg, France), WWTP (Ceuta, Spain), spring water (Guaro, Periana, Spain) and tap water (Málaga, Spain). The samples were collected in polypropylene bottles (previously cleaned by soaking for 24 h in 10% (v/v) nitric acid and finally rinsed thoroughly with ultrapure water before use. The samples were immediately filtered using a cellulose nitrate membrane of 0.45 mm pore size from Millipore (Bedford, MA, USA). After filtration, the samples were acidified to 0.1% (wt/wt) by the addition of concentrated HNO_3_ and were stored in low density poly-propylene bottles at 4º C, as recommended by Method 3010B from the Environmental Protection Agency (USA), for less than 3 days until analysis. For the analysis of these samples, aliquots were placed in volumetric flasks; the pH was adjusted to 5 using an acetic-acetate buffer (10% of the final volume). The samples were spiked when required and finally, DDW was added up to the mark.

### Online measurement protocol

**Fig. 1:**
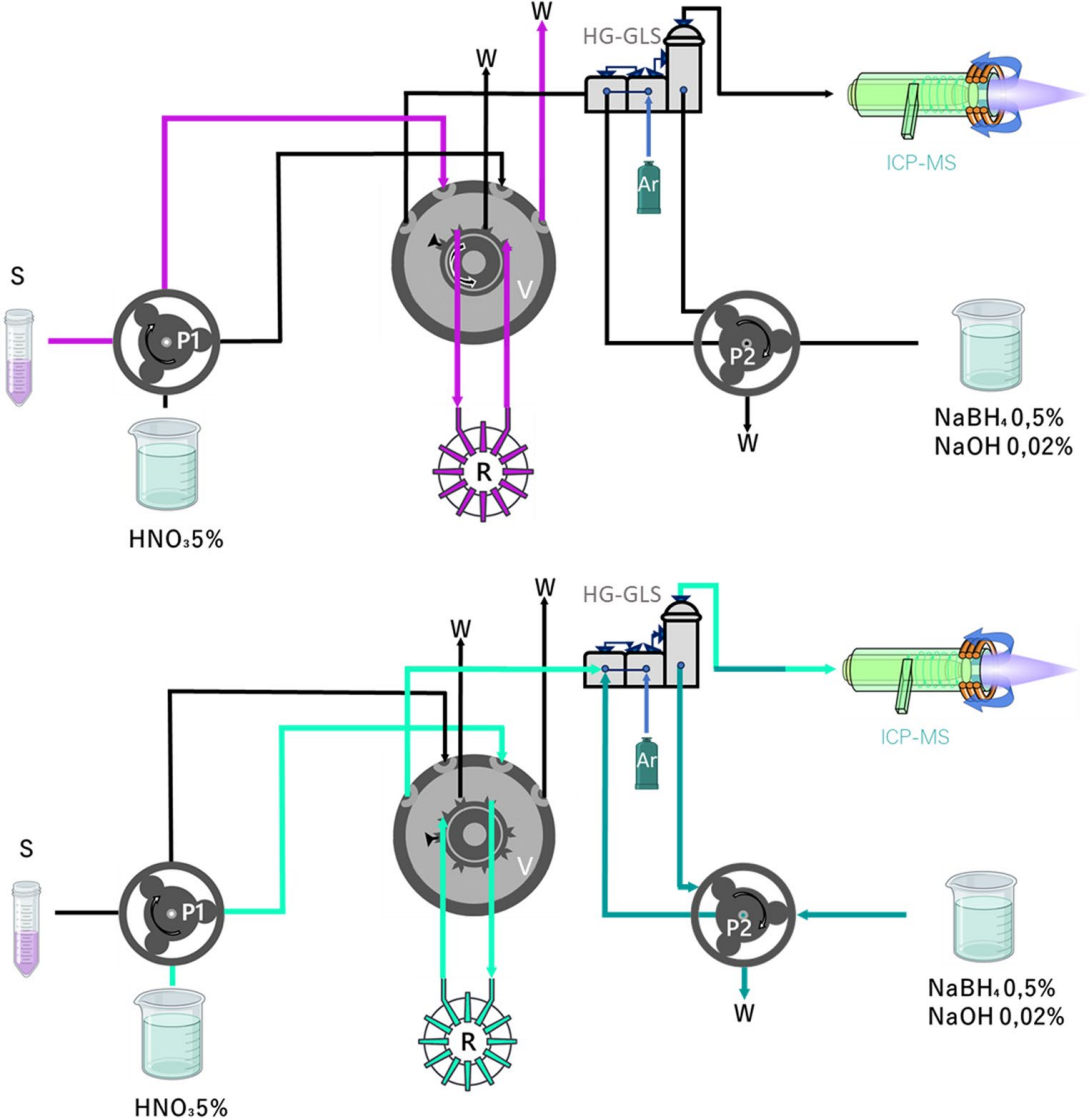
MSPE-FI-HG-GLS-ICP-MS scheme in load position (A) and elution position (B). P1 and P2, peristaltic pumps. S, sample. V, valve. W, waste. R, knotted reactor with M@GONIO inside

The MSPE-FI-HG-GLS-ICP-MS system is schematically illustrated in Fig. 1. This protocol consisted of two steps: the first step, with the five-port valve in A position, and second step, with the valve in B position. In the first step, pump 1 (P1) loaded sample into the reactor during 120 s at 5 mL min^− 1^, the remaining solution flowed to waste. In the second step, the autosampler introduced DDW into the reactor for 30s at 4mL min^− 1^, to remove the excess matrix from the reactor. Then, the valve was switched to position B where 5.5% HNO_3_ was pumped at 3.5 mL min^− 1^ through the reactor in reverse flow relative to the preconcentration step (avoiding a continuous increase in the compactness of the reactor). This eluent flow was directed to the HG where it was mixed with the reductant solution of NaBH_4_ and NaOH (pumped with P2 at 0.8 mL min^− 1^) and with an Ar carrier gas. Thus, the hydride reaction took place, producing SbH_3_ and AsH_3_, which were transported through the hydrophobic membrane and directed to the ICP-MS by a stream of Ar flow at 0.9 L min^− 1^. Signals were recorded for 99 s.

The remaining liquid phase was extracted and send out to waste by P2 at 3.5 mL min^− 1^ and the reactor was ready for the next sample loading. This process was repeated three times, corresponding to the number of replicates defined in the analytical method.

### Optimization strategy

The methodology was optimized to achieve the best performance. This optimization procedure can be divided into three groups, instrumental, chemical, and FIAS parameters. The instrumental parameters are related to the ICP-MS performance, which was mostly optimized daily by the SmartTune-Syngistyx software. The chemical parameters, include the MSPE conditions (pH of the sample, reductant concentration, eluent composition and concentration) and the FIAS conditions, include flow-rates of each stream, number of steps and time of each step.

Two optimization strategies were employed: a univariate approach, carried out by changing one parameter at a time, with the remaining parameters kept constant., as the pH optimization (described in Sect.  2.2.), and a multivariate strategy based on the use of a central composite design (CCD) with multiple responses. A central composite design plus a star was the response surface design chosen to optimize the FIAS parameters (flow-rates of loading and elution steps and eluent concentration), where 2^k^ + 2·k + n runs were generated, being k the parameters to optimize (k = 3) with two responses (signal/noise from As and Sb measurements). The repetition of the central point was used to estimate the experimental error (*n* = 2) in the design. For this optimization, a standard containing 100 ng L^− 1^ As and 50 ng L^− 1^ Sb and a blank were employed. The response functions were the signal-to noise ratio for ^75^As and ^121^Sb, with the best S/N ratio for both analytes being the optimization criterion (desirability) in this study. The minimum and maximum values introduced for the CCD study were 0.6 and 5.0 mL min^− 1^ for loading flow-rate; 0.6 and 3.5 mL min^− 1^ for elution flow-rate and 3 and 8% wt/wt for HNO_3_ concentration.

Statgraphics Centurion software (version 16.1.11 for Windows) was used for processing the experimental data. Analysis of the variance (ANOVA) and p-value (the probability of the effect of a factor being due solely to random error) significance levels were used for checking the significance of the effects in the design.

### Synthesis of the nanomaterial

M@GO was synthesized by coupling GO and MNPs as reported in our patent [22]. GO was obtained by the chemical oxidation-exfoliation of graphite [23] and MNPs were obtained by coprecipitation of Fe^2+^ and Fe^3+^ in ammonia media.

The functionalization was carried out in a sealed tube, where 100 mg of M@GO were suspended in 4.5 mL of acetonitrile and 1.5 mL of iodomethane were added. The mixture was heated overnight at 80 °C. Then, the solid was magnetically decanted and washed with DDW, 1% NaOH and ethanol; the material was then dried for 72 h.

**Fig. 2:**
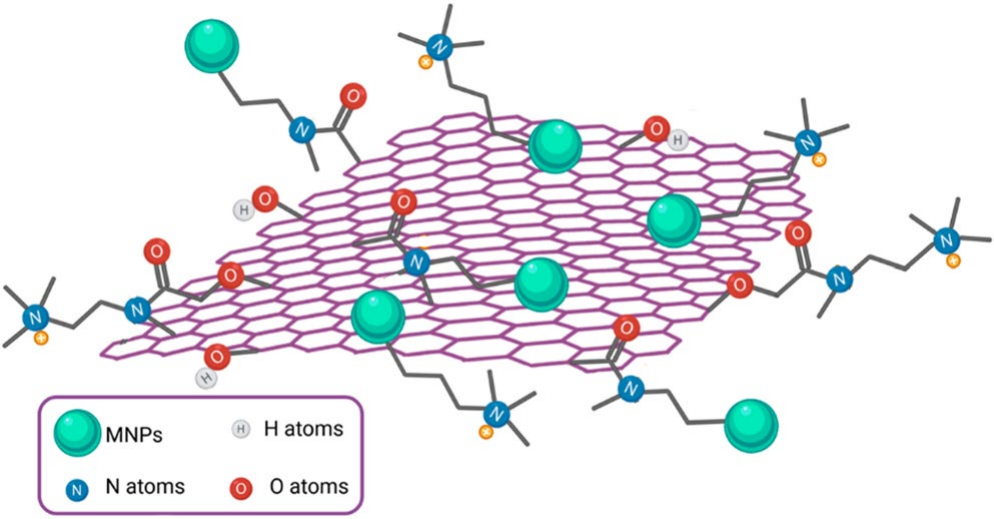
M@GONIO structure

The final structure of the new nanomaterial is shown in Fig. 2, which presents ammonium groups with a positive charge, useful for metal chelation.

### Isotherm model

Additionally, a study of the adsorption performance over a wider range was developed to determine the adsorption isotherm of these analytes on M@GONIO. The removal of As and Sb from water was studied by batch experiments, mixing 5 mg of the nanomaterial with 50 mL of different solutions buffered at pH 5 with different concentrations of As and Sb in the range from 80 µg L^− 1^ to 2000 µg L^− 1^. They were mixed on an end-over-end shaker at 50 rpm for 72 h.

Afterward, the phases were magnetically separated and 15 mL of 5.5% (wt/wt) HNO_3_ was added to the nanomaterial in order to preconcentrate and extract the ions. The extraction was carried out by ultrasonication for 10 min, following the phases were magnetically separated, and the eluent was measured directly by ICP-MS. The concentration in the supernatant was calculated by mass balance. Prior to adsorption experiments, the equilibrium time was determined using six samples with initial concentrations of 80 µg L⁻¹ Sb and 160 µg L⁻¹ As. These samples were treated following the same procedure described above, with continuous mixing for 6 h. The amount of adsorbed analyte was measured at 1 h intervals until adsorption saturation (equilibrium) was reached.

The adsorption isotherm obtained at 23 °C was fitted to the most appropriate isotherm model, and the corresponding parameters were determined.

## Results and discussion

### Characterization and performance of M@GONIO

The nanomaterial was characterized using several techniques, which demonstrated that the functionalization reaction was properly achieved.

#### **SEM&TEM**

**Fig. 3:**
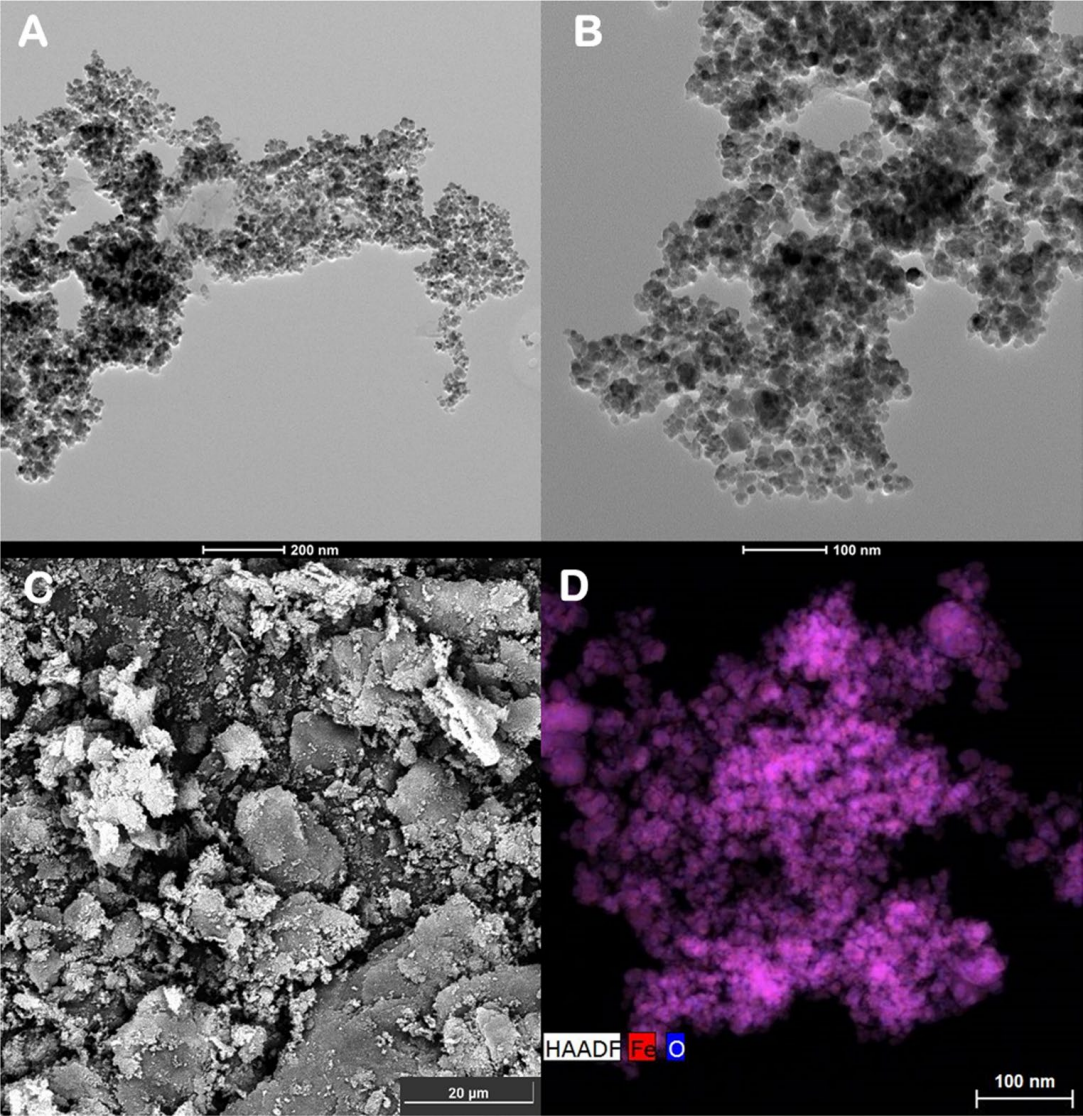
SEM&STEM images of M@GONIO. (**A**) TEM scale 200 nm, (**B**) TEM scale 100 nm, (**C**) SEM scale 20 μm and (**D**) HAADF scale 100 nm

Surface morphology of the nanomaterial was observed with these microscopy techniques, as shown in Fig. 3, MNPs, with a diameter between 10 and 20 nm, were dispersed randomly on the surface of GO nanosheets. Figure 3D shows a high-angle annular dark-field scanning transmission (HAADF) image. The dispersion of the MNPs can be observed, as Fe and O atoms are distributed across the entire surface of nanosheets.

#### **Elemental analysis**

This analysis revealed the presence of C, N, H and O with weight percentages (wt/wt) of 26.88; 1.61; 3.89 and 26.83%, respectively, confirming the existence of nitrogen-derived functional groups.

#### **XPS analysis**

**Fig. 4:**
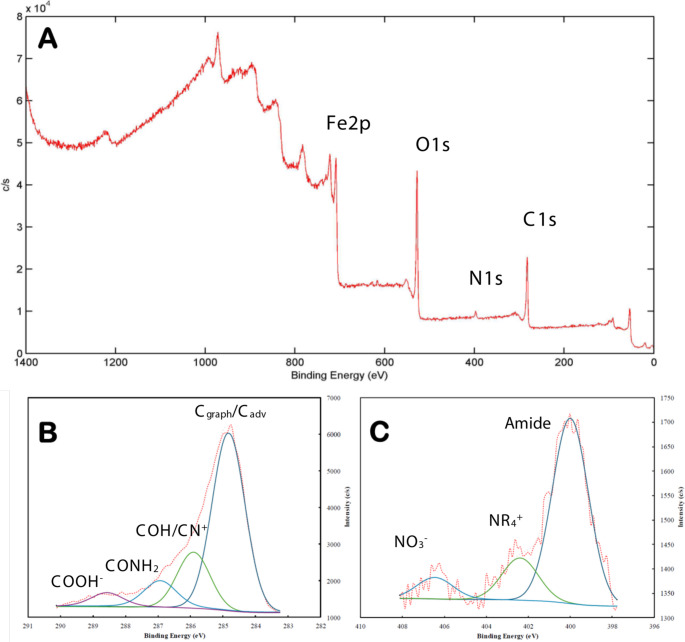
XPS results. (**A**)Full spectrum, (**B)** C(1s) region and (**C**) N (1s) region (Reference peak C^adv^ at 284.8 eV, pressure during data acquisition 3·10^− 9^ Torr)

XPS spectra are related to the surface composition of the first layers of the nanomaterial. In Fig. 4, the entire spectrum can be observed, where C, Si, Fe, O and N elements, characteristics of M@GONIO composition, are shown; atomic percentages obtained for C, O, N, Si, and Fe were 41.99; 37.37; 3.55; 2.58 and 14.51% respectively. The N(1s) region was deconvolutioned, Fig. 4C, revealing three peaks: 400.0 eV corresponding to amide groups, 402.6 eV peak assigned to quaternary alkylammonium groups, and 406.4 eV peak attributed to nitrate groups related to the surface oxidation [24]. XPS data confirmed the presence of GO through the C and O content, Fe and Si confirmed the MNPs presence and quaternary N corroborated the successful functionalization of the material.

#### **FT-IR**

**Fig. 5:**
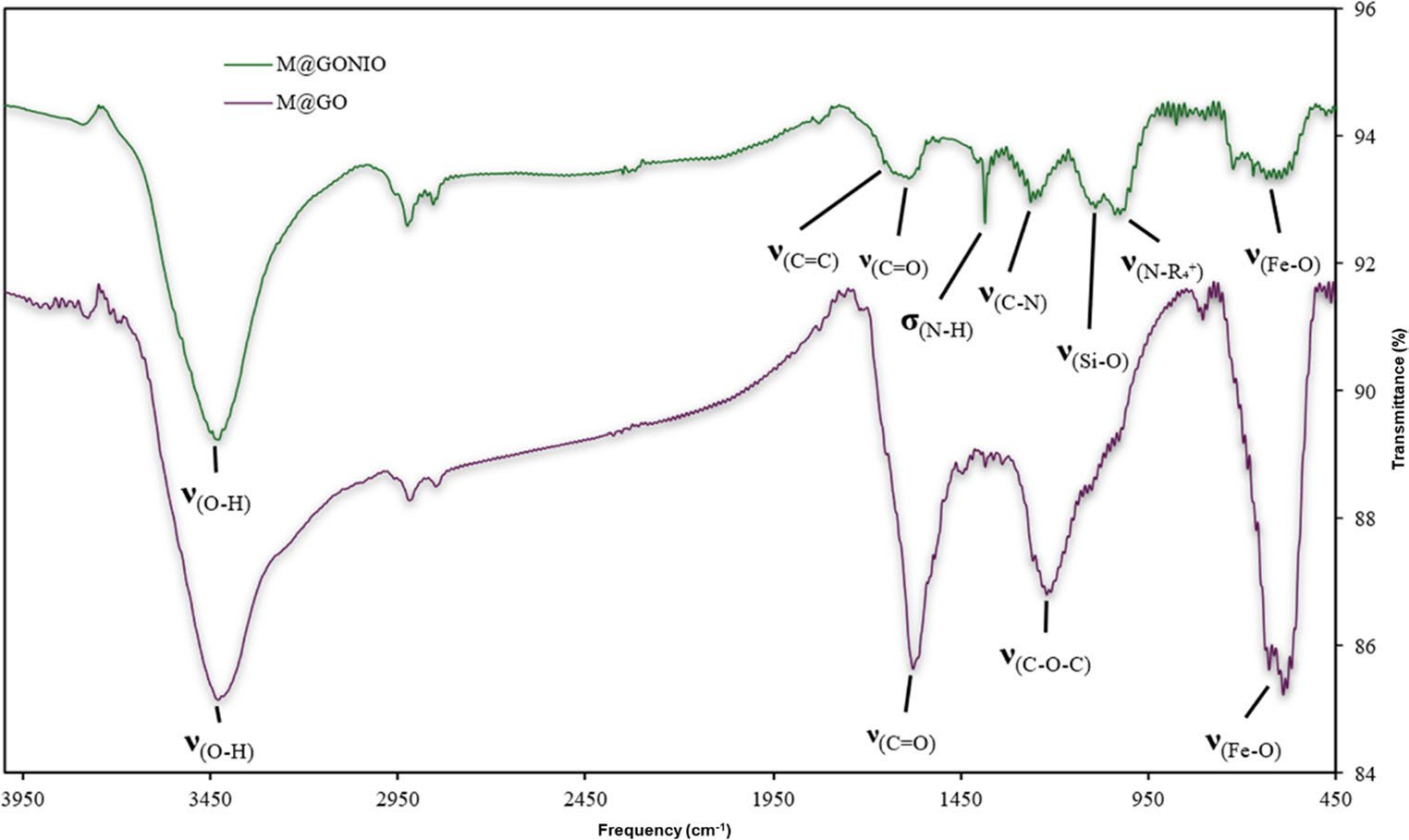
FTIR spectrum of M@GONIO and M@GO (KBr pellets)

The FT-IR spectra interpretation is complex due to the aromatic contributions in the nanomaterial, mostly from GO, which generates numerous overlapping bands. However, certain assignments can be made in the mid infrared region. Figure 5 shows the similarities and differences between the FT-IR spectrum of M@GO and M@GONIO. Some bands corresponding to the functional groups introduced during the functionalization of M@GONIO are observed, such as the overlap observed between amide I and the aromatic skeletal bands in the region 1550–1650 cm^− 1^. The σ_N−H_ amide II and the νC−N amide III bands are observed at 1380 and 1250 cm^− 1^ respectively, these bands along with the quaternary ammonium band at 1020 cm^− 1^ confirm the successful functionalization of the material. The bands at 1090 cm^− 1^ and 600 cm^− 1^ correspond to and ν_Si−O_ and ν_Fe−O_ respectively, suggest that the dispersion of iron nanoparticles in GO has occurred properly. These spectral features confirm the synthesis and functionalization of the nanomaterial.

#### **Nitrogen adsorption/desorption isotherms**

**Fig. 6:**
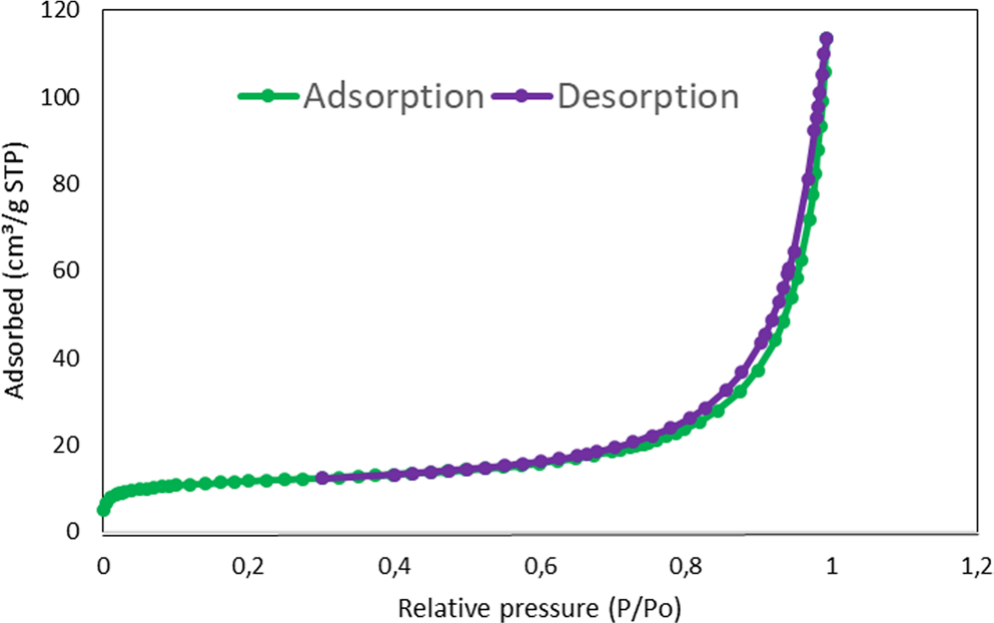
N_2_ adsorption/desorption isotherms (EI = 20 s)

The nitrogen adsorption-desorption isotherm is shown in Fig. 6 which resembles type IV isotherms, commonly found for mesoporous materials (pore size between 20 and 500 Å) [22, 23]. For this material, the pore size was found to be 79 Å and the surface area obtained was 42.2 m^2^ g^− 1^. As can be observed from the pore size, the nanomaterial can be classified as mesoporous and presents a higher surface area than GO (2.63 m^2^ g^− 1^).

#### Mass spectrometry

The characteristic peaks observed in the MS spectra were assigned to different fragments. The most relevant peaks are indicated in Table 2.

**Table 2:**
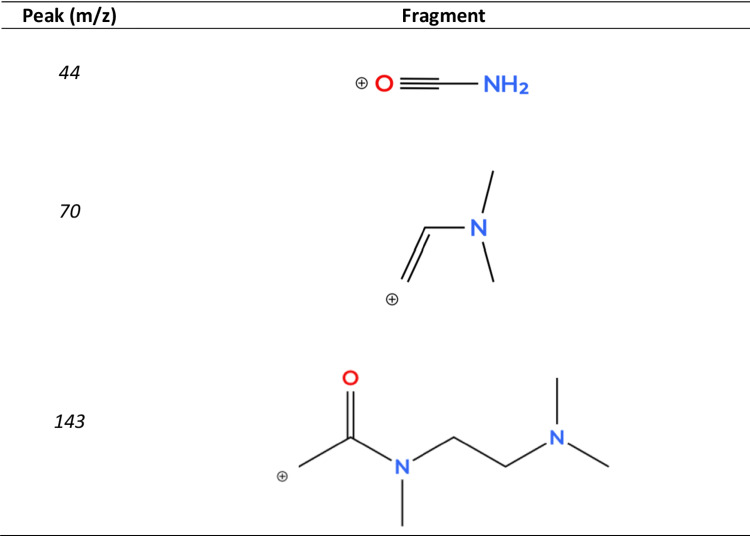
MS identification results for some fragmentations (RT=0.93 and NL=1.06E7)

#### **Isotherm model of the nanosorbent**

**Fig. 7:**
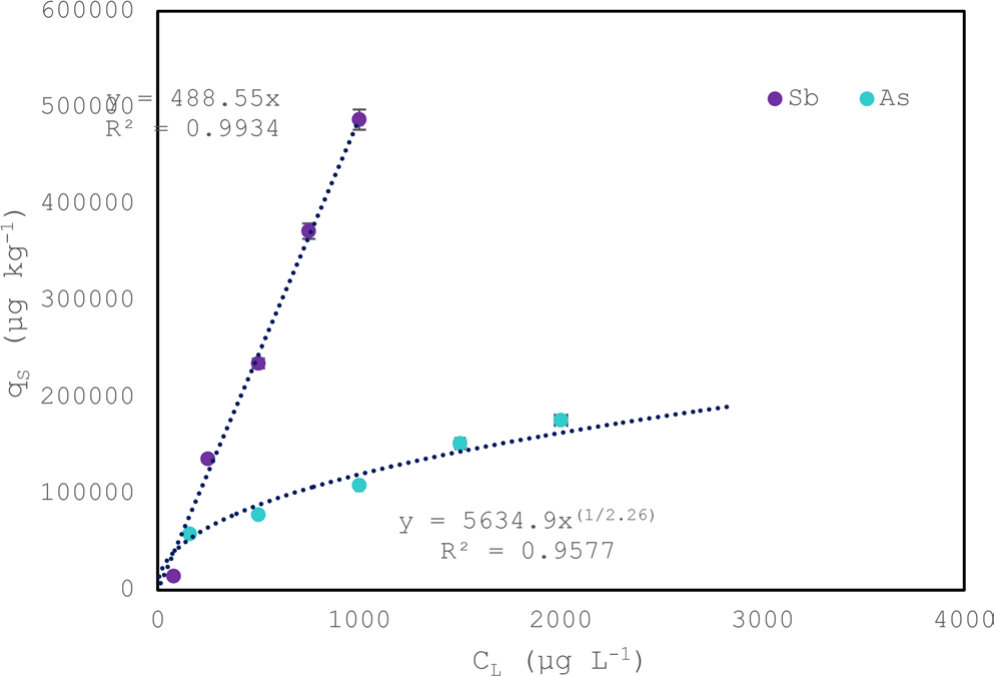
Equilibrium studies for As and Sb adsorption on M@GONIO

After the determination of the equilibrium time for both analytes (3 h for Sb and 6 h for As), different initial concentrations were tested, and their adsorption performances were studied in the equilibrium. The adsorption capacity of As and Sb on M@GONIO is shown in Fig. 7, where the analyte concentration adsorbed on the nanomaterial (q_S_) is plotted against the analyte concentration in the supernant (C_L_); The horizontal error bars represent the standard deviation associated with the liquid-phase concentration, and the vertical bars correspond to the uncertainty in the solid-phase concentration.

For Sb, the experimental data exhibit a linear relationship that passes through the origin, indicating that the adsorption process can be described by a linear adsorption isotherm shown in Eq. 1:

1 $$q_S=K_d\cdot C_L$$

where q_S_ is the adsorbed amount at equilibrium in µg/kg, C_L_ is the equilibrium concentration in solution in µg/L, and K_d_ is the adsorption equilibrium constant in L/kg. The obtained equilibrium constant for Sb was K = 488 L/kg, suggesting a proportional partitioning of Sb between the aqueous phase and the adsorbent within the studied concentration range. Such linear behaviour is typically observed at low surface coverage, where the number of available adsorption sites greatly exceeds the number of adsorbed species. Under these conditions, adsorption sites behave independently, resulting in negligible interactions between adsorbed molecules and a proportional increase of q_S_ with increasing C_L_ [25, 26].

In contrast, the adsorption data for As do not follow a linear relationship passing through the origin. Instead, the experimental points are better described by the Freundlich isotherm model, Eq. 2:

2 $$q_S=K\cdot C_L{}^{1/n}$$

where K is the Freundlich adsorption constant and n is an empirical parameter related to adsorption intensity. The fitted parameters were *n* = 2.26 and K = 5635, where the value of K corresponds to q_S_ expressed in µg/kg and C_L_ in µg L⁻¹. The nonlinear behaviour observed for As suggests a more heterogeneous adsorption process [27], likely associated with differences in adsorption site energies or stronger interactions between arsenic species and functional groups present on the surface of M@GONIO.

### Optimization procedure

Chemical and FIAS parameters optimization was carried out with two possible procedures, univariate and multivariate. The latter was approached as multiple response central composite designs in order to obtain the most beneficial conditions for all the parameters involved in the simultaneous determination.

#### Effect of pH

Since the functional groups in the nanomaterial are affected by pH, the interaction with ions strongly depends on this parameter, so it was the first one to be optimized. Standard solutions of 10 µg L^− 1^ of each target analytes were prepared and buffered to the adequate pH for each analysis, as described in Sect.  2.2.

**Fig. 8:**
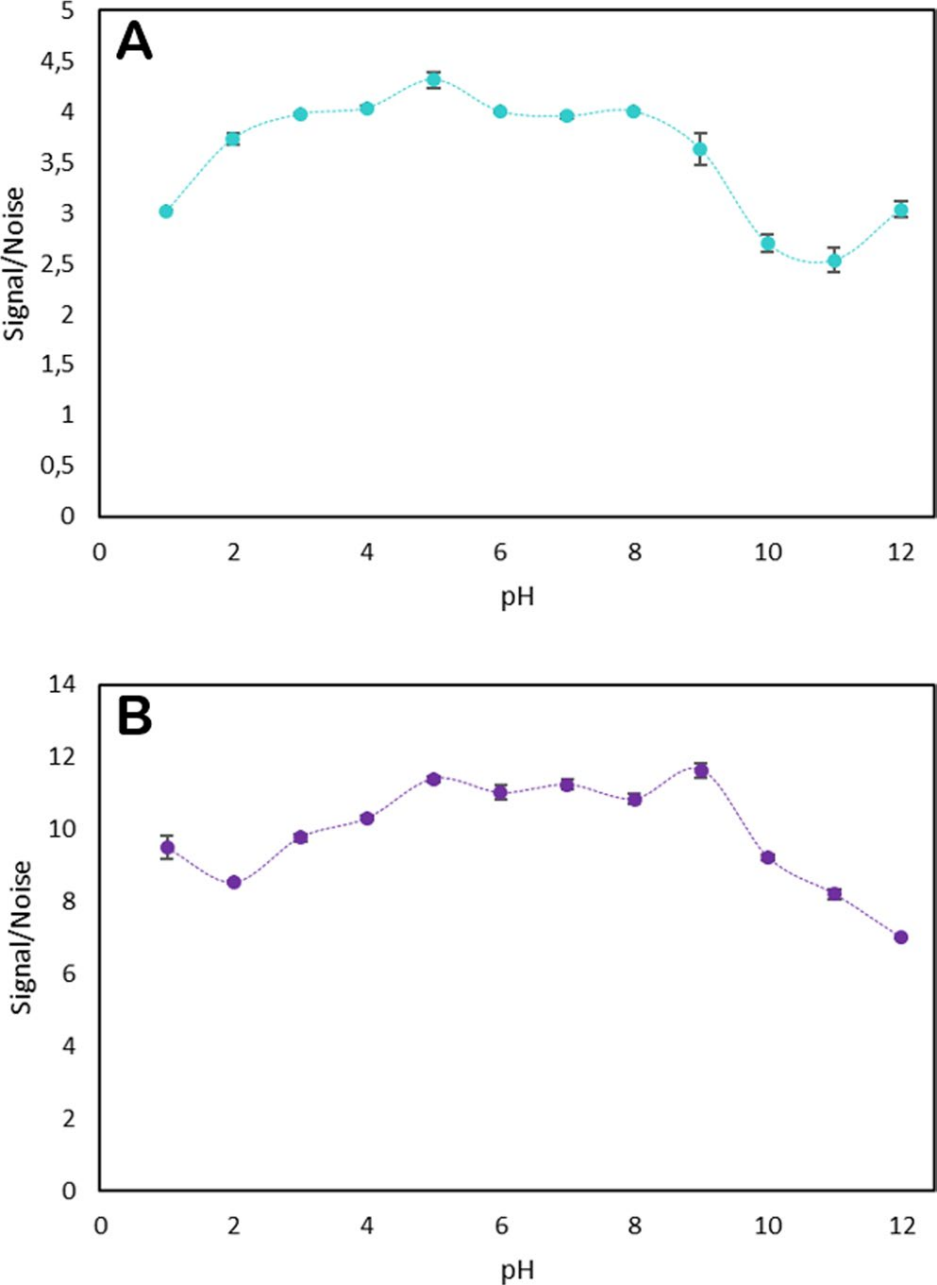
Study of pH in the samples As (**A**) and Sb (**B**)

The results obtained for each analyte (Fig. 8) show similar behaviour, as expected. Quantitative adsorption appears to occur in the pH range from 5 to 9, with a maximum for As at pH 5 and for Sb at pH 9 (Fig. 8A and B).

This range corresponds to the typical pH range of environmental waters; therefore, samples could be analyzed without the use of buffer solutions. In the typical pH range of natural waters (pH 5–9), As(V) is present as H₂AsO₄⁻/HAsO₄²⁻, and Sb(V) predominantly occurs as Sb(OH)₆⁻. Under these conditions, both elements are predominantly present as oxoanions, which explains their similar adsorption behavior on quaternary ammonium-based adsorbents, used in this work. Since comparable extraction efficiencies were obtained throughout this pH range, pH 5 was finally selected as the working condition because it provided slightly better signal-to-noise ratios for As determination, and satisfactory recoveries for both analytes were maintained.

#### Effect of eluent

**Fig. 9:**
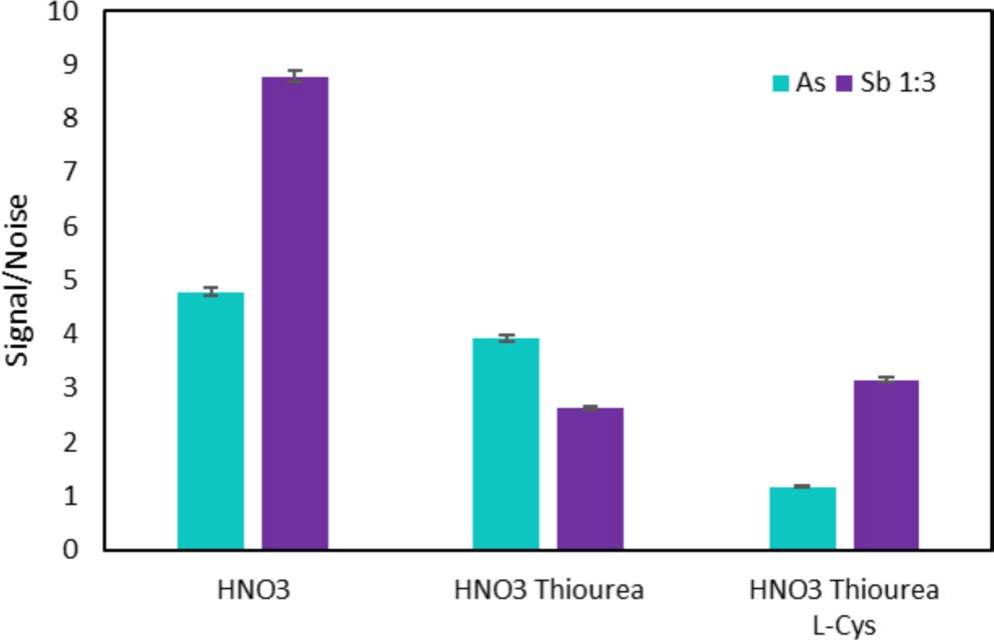
Study of the eluent effect

The elution step is critical for the preconcentration process, as achieving maximum analyte recovery is essential for optimal analytical performance. At pH < 2, both As and Sb are predominantly present as neutral species. Specifically, As(V) and As(III) occur mainly as H₃AsO₄ and H₃AsO₃, respectively, and Sb(V) and Sb(III) are present as Sb(OH)₅ and Sb(OH)₃. Under these strongly acidic conditions, the oxoanion character of both elements is lost, resulting in negligible retention on anion-exchange adsorbents, such as the quaternary ammonium–type materials used in this work. In addition, the formation of their volatile hydrides also requires a reducing environment. For both reasons, these media were evaluated as eluents. Thiourea (Ty) and L-cysteine (L-Cys) are commonly used as pre-reductants agents [28–31]. Three eluents were tested: HNO_3_ 5%; HNO_3_ 5%-Ty 0.04%; and HNO_3_ 5% Ty 0.1%-L-Cys 2.8%. As shown in Fig. 9, the results indicated that the highest performance for both analytes was achieved using HNO₃ as the sole eluent; however, the signal-to-noise (S/N) ratios were consistently higher for Sb due to significant blank contributions observed for As. In Fig. 9, the Sb results are presented using a 1:3 scaling of the obtained values to allow better fitting and comparison of both analytes in the graph.

Finally, the optimum HNO_3_ concentration was studied together with FIAS parameters by applying a Box-Behnken CCD study.

#### FIAS parameters optimization

The optimization of flow-rates in the system is crucial for the method performance and the efficient preconcentration of the analytes. Since the volume of eluent affects directly the preconcentration factor, the higher enrichment factors (EF) are obtained at lower elution flow-rates. Conversely, loading flow must ensure a efficient adsorption of the analytes, ideally in a short period of time, as it influences recovery ratios and the analytical performance.

**Fig. 10:**
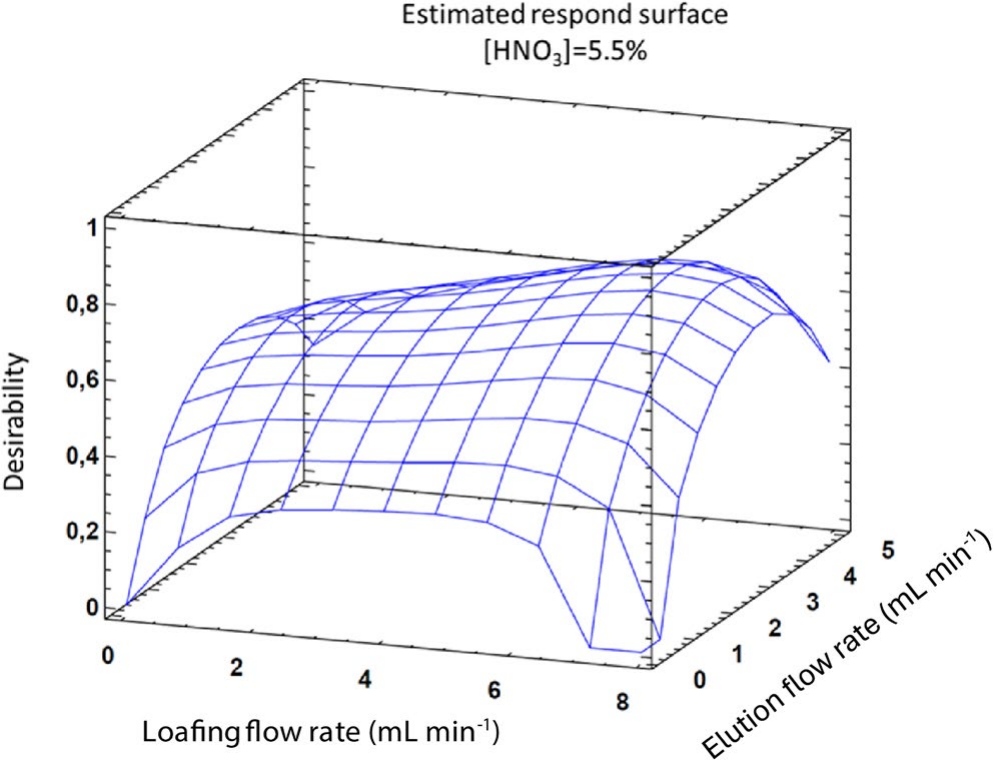
Response surface obtained for both analytes

Sample and eluents flow-rate effects were studied in combination with HNO_3_ (eluent) concentration applying a Box-Behnken CCD involving 16 runs to determine the optimal conditions, as described in the previous 3.2.2 section. Based on the results provided by the Statgraphics software, Fig. 10, the optimal conditions obtained were 6.5 mL min^− 1^ for loading flow-rate and 3.5 mL min^− 1^ for elution flow-rate. This optimization was performed considering a constant sample volume (3 mL) to ensure comparability, as larger volumes would increase signal intensity; consequently, the loading time was adjusted for each flow-rate condition.

Although the optimized loading flow-rate exceeded the elution flow-rate, which is favourable for obtaining a high preconcentration factor, it approached the operational limit of the FIAS system. To avoid potential overpressure risks to the system, a loading flow-rate of 5 mL min^− 1^ was selected as optimum for sample loading.

Following the loading step, the dead volume inside the reactor, occupied with residual matrix components, must be removed. A washing step using DDW was therefore introduced. Washing time between 30 and 90 s and flow-rates between 2.5 and 6 mL min^− 1^ were studied A washing time of 30 s was sufficient to clean the reactor, providing the highest signals for both analytes. Finally, 4 mL min^− 1^ was the best flow-rate for the washing step.

Efficient waste removal from the HG-GLS system is essential to ensure the correct hydride formation, as liquid accumulation may wet the membrane and hinder gas-phase transmission. In this set-up, the GLS system waste and elution flow-rates are controlled at the same time by pump P2. Since the elution flow-rate is crucial for the performance of the method, this parameter was the first parameter optimized. With respect to the waste flow rate, excessively high flow rates may entrain hydrides, however, insufficient flow rates may not adequately remove the liquid, leading to membrane wetting and subsequent blockage of hydride transport to the ICP-MS. Under the optimized conditions, waste flow-rate was 3.5 mL min^− 1^ which provided efficient HG-GLS operation and produced high signals for both analytes.

#### Effect of loading time

The preconcentration (loading) time is a crucial parameter for the analytical performance of the method. The enrichment factor is directly related to the preconcentration time, which was studied between 1 and 5 min, keeping the remaining parameters at their optimal values previously optimized. A linear increase in signal was observed for both analytes until five minutes. As expected, longer loading times resulted in higher signals, and consequently, improved sensitivity. However, increasing the loading time reduces the sampling frequency and leads to higher costs, as well as greater consumption of samples and reagents. Therefore, a loading time of 2 min was selected as a compromise between high sampling frequency and adequate sensitivity, though longer loading times may be applied when required by low analyte concentrations in the samples.

#### Hydride generation optimization

Both analytes are introduced into ICP-MS as hydrides, consequently, the step required to generate these gases is critical, and the parameters involved must also be optimized. Reductant concentration was studied in a range of 0.05–4% (wt/wt) for NaBH_4_ (with NaOH kept at 20% relative to the NaBH₄ concentration to ensure solution stability). Regarding As, the best results were achieved with 0.05% NaBH_4_; and Sb showed better signals using 0.5% NaBH_4_. Given this situation, 0.5% (wt/wt) was the concentration selected because As had the second-best performance at this NaBH_4_ concentration and Sb showed the best performance. Regarding flow-rate, 0.5, 0.8 and 1 mL min^− 1^ were tested, and 0.8 mL min^− 1^ achieved better signals for both analytes.

### Reuse of the nanomaterial

Successive loading/elution cycles were carried out to evaluate the reusability of the nanosorbent with the configuration and conditions previously described, using a standard of 10 ng L^− 1^ for both analytes and pH 5. The reusability and usability of the reactor was established to exceed 200 cycles of measurement, after which an obvious signal loss was observed. The low consumption of sample and reagent fits the proposed method within the principles of Green Analytical Chemistry.

### Analytical performance

Under the optimal conditions obtained, the performance of the method was studied for a preconcentration time of 2 min. The analytical features including calibration curves, limits of detection and quantification, enrichment factor and precision are summarized in Table 3. A linear calibration was obtained for both analytes. The limits of detection (LOD) and limits of quantification (LOQ) were defined as $\overline{x}$ + 3s (*n* = 11) and $\overline x$ + 10s (*n* = 11) respectively; where $\overline x$ is the mean of the blanks and s their standard deviations, obtaining LODs of 9.4 ng L^− 1^ and 0.38 ng L^− 1^ for As and Sb, respectively; and LOQs of 25.6 ng L^− 1^ and 5.82 ng L^− 1^ for As and Sb, respectively, with a wide linear range, from the LOQ to, at least, 100 µg L^− 1^. The inter-day and intra-day precision of the method (RSD) was determined as the relative standard deviations of standards of 50, 500 and 1000 ng L^− 1^ for As and 10, 50 and 100 ng L^− 1^ for Sb and the enrichment factor (EF) was defined as the ratio between the slopes of two calibration curves, one obtained, employing the reactor containing the nanomaterial, and another one obtained employing an empty reactor (one with preconcentration and one without). If necessary, detection and determination limits and EF can be improved by employing longer preconcentration time, however, with 2 min loading time, a complete cycle of measurement requires 250 s per sample, allowing the analytes to be determined with a throughput of 14.4 h^− 1^.

**Table 3:** Analytical features

Analyte	Calibration graph	Blank ± S_b_	LOD (ngL^− 1^)	LOQ (ngL^− 1^)	EF	RSD (%)^a^ *N*=7	RSD (%)^b^ *N* = 7	RSD (%)^c^ *N* = 7	RSD (%)^d^ *N* = 5
As	y = 53.86x + 2943.9	3077 ± 125	9.4	25.6	84.7	2.1	1.6	3.3	3.1
Sb	y = 1289.6x + 20,322	17,802 ± 1003	0.38	5.82	84.6	3.7	2.5	5.1	3.2

^a^RSD intraday: As 50 ngL^− 1^ and Sb 5 ngL^− 1^

^b^RSD intraday: As 500 ngL^− 1^ and Sb 50 ngL^− 1^

^c^RSD intraday: As 1000 ngL^− 1^ and Sb 500 ngL^− 1^

^d^RSD interday: As 500 ngL^− 1^ and Sb 50 ngL^− 1^

In order to compare the performance of the method, a study of the state of the art for similar methods was carried out (Table 4). Since few methods report the simultaneous SPE and quantification of both As and Sb, methods determining only one of them have also been considered. This comparison is difficult due to the differences in the experimental conditions among the methods, but some relationships and conclusions can be obtained. From the data summarized in Table 4, it can be concluded that the method developed in this work provides relative standard deviations comparable to those reported in the literature. In addition, the enrichment factors and the limits of detection and quantification achieved for As are among the best reported. For Sb, the proposed method exhibits the highest sensitivity. Furthermore, the method operates in an online and fully automated mode, which makes it particularly suitable for the routine determination of trace levels of these pollutants in environmental waters.

**Table 4:** State of the art comparison

Analyte	Adsorbent	LOD (ngL^-1^)	LOQ (ngL^-1^)	EF	RSD (%)	Ref
Sb	ZIF-8	70	-	27	3.8	[32]
As	SWCNTs	3.8	-	25.4	4.2	[33]
Sb	SWCNTs	2.5	-	24.6	4.8	[33]
As	Modified mesoporous TiO_2_	530	-	-	3.9	[34]
Sb	Modified mesoporous TiO_2_	770	-	-	5.2	[34]
As	Eggshells	1	-	33.3	2.1	[35]
Sb	SiO_2_/Al_2_O_3_/SnO_2_	170	560	136	-	[36]
As	Modified activated C	50000	-	50	4.1	[37]
Sb	IIP-Fe_3_O_4_@SiO_2_@CNFs	130	440	71.3	4.7	[38]
As	C nanotubes	30	-	75	3.1	[39]
Sb	C nanotubes	60	-	75	3.5	[39]
As	M@GONIO	9.4	25.6	84.7	2.1	This work
Sb	M@GONIO	0.38	5.82	84.6	3.7	This work

### Analytical application

Once the method was optimized, it was employed for the analysis of certified reference samples, TMDA 64.3 and SPS-WW2, and real samples (tap, sea, river, canal, WWTP, and spring waters). External standard calibration was employed for these analyses, The results, summarized in Table 5, demonstrate the high accuracy of the proposed method. Besides, in Table 5 are included recovery assays performed as an additional validation process; high recoveries close to 100% were obtained for all the spiked samples confirming the accuracy of the method even for complex matrices. The content of both analytes in drinking water obtained is consistent with the WHO permitted limits.

**Table 5:** Analytical application

Sample	Certified (µgL^-1^)	Added (µgL^-1^)		Found (µgL^-1^)		Recovery (%)	
		As	Sb	As	Sb	As	Sb
TMDA 64.3	163±13 (As) 124±10 (Sb)	-	-	177±8	122±4	-	-
		109	124	280±10	244±6	97.9	99.2
		272	290	450±3	380±20	100.2	92.2
		543	579	720±50	726±3	100.0	103.6
SPS-WW2	500±3 (As)	-	-	520±20	64±3	-	-
		333	166	850±30	228±8	99.6	99.1
		833	333	1330±30	410±10	98.3	103.3
		1666	833	2190±90	920±4	100.2	102.6
Ceuta WWTP	50±5 (Sb)*	-	-	10.0±0.4	46.0±0.5	-	-
		16.7	16.7	25.8±0.6	63±2	96.6	100.5
		33.3	33.3	44.6±0.8	76.6±0.3	103.0	96.6
		66.6	66.6	81±3	106.4±0.9	105.7	94.5
Tap water	-	-	-	8.5±0.4	4.0±0.1	-	-
		10	10	17.8±0.8	13.4±0.3	96.2	95.7
		20	20	27.1±0.4	24.3±0.9	95.1	101.3
		40	40	50.3±1.4	45±2	103.7	102.3
Tinto River	-	-	-	57.0±1.1	13.17±0.14	-	-
		10	5	67±2	18.2±0.4	100.0	100.2
		20	10	76±3	23.8±0.2	98.7	102.9
		40	20	101±3	33.6±1.1	104.1	101.3
Almería sea water	-	-	-	5.56±0.18	2.01±0.03	-	-
		5	5	10.0±0.4	7.34±0.02	94.7	104.7
		10	10	15.5±0.3	11.2±0.1	99.6	93.3
		20	20	26.7±0.2	22.3±0.7	104.5	101.3
Guaro spring water	-	-	-	7.23±0.09	9.02±0.09	-	-
		5	5	12.1±0.3	13.7±0.6	98.9	97.7
		10	10	16.53±0.02	19.9±0.3	95.9	104.6
		20	20	25.8±0.3	29±1	91.4	99.9
Alcaucín river	-	-	-	4.75±0.15	20.2±0.9	-	-
		5	5	10.2±0.3	25±1	104.6	99.2
		10	10	14.5±0.3	30.5±0.9	98.3	101.0
		20	20	24.4±0.7	40.4±0.5	98.6	100.5
Strasbourg canal	-	-	-	3.85±0.04	30.2±0.3	-	-
		5	5	8.96±0.14	36.1±1.1	101.2	102.6
		10	10	13.5±0.1	41.0±1.4	97.5	102.0
		20	20	25.8±0.8	50.0±1.8	108.2	99.6
		50	50	56±2	86±2	104.0	107.2

*Analysed at Research Supporting Central Services of University of Málaga (SCAI-UMA)

Moreover, as the certified samples included diverse trace elements that did not affect the method performance and accuracy, it can be concluded that there are no interferences from these metals at similar concentration levels as the analytes. Although the material may potentially interact with other oxyanions through electrostatic interactions, the combination of the proposed extraction methodology with the HG-ICP-MS detection system provides adequate selectivity and reliable determination of the target analytes at ultratrace concentration levels. Similarly, based on sea water samples, which have noteworthy salinity, chloride (a well-known interference for ^75^As due to the ^40^Ar^35^Cl specie formation) did not interfere with arsenic determination, as expected when using HG.

Analysing real samples results, several conclusions can be obtained. First, as expected from Tinto River (one of the most contaminated rivers in the Iberian Peninsula), the levels of both analytes are high and exceed WHO limits due to the historical mining activities in the area [40]. Also related to mining activities, levels found in Strasbourg can be related to antimony mines in Sulzburg (Germany) and Goesdorf (Luxemburg) which are connected to Strasbourg through Rhine, Sauer and Moselle rivers [41]; similarly, Sb levels in Alcaucín can be explained by native antimony naturally present in this area, which has even been exploited in mining activities at Matilde mine, previously called La Victoria mine (La Viñuela) [42–44]. On the other hand, the source of Guaro River, also close to Alcaucín, presents levels under WHO limits. Almería soil presents one of the highest As levels in Spain [45] consequently, As in Almería southwest coast has presented high levels too, in this sample analysed from the east coast; As concentration is higher than Sb.

### Greenness assessment using the AGREE metric

The environmental performance of the proposed analytical method was evaluated using a multi-criteria greenness assessment tool based on ten sustainability indicators. The resulting radial diagram provides a comprehensive visual overview of the environmental profile of the analytical workflow. Each sector corresponds to an individual evaluation criterion, where the color scale from green to red indicates the degree of compliance with green analytical chemistry principles, green represents a more sustainable performance and red highlights aspects with a higher environmental weight.

**Fig. 11:**
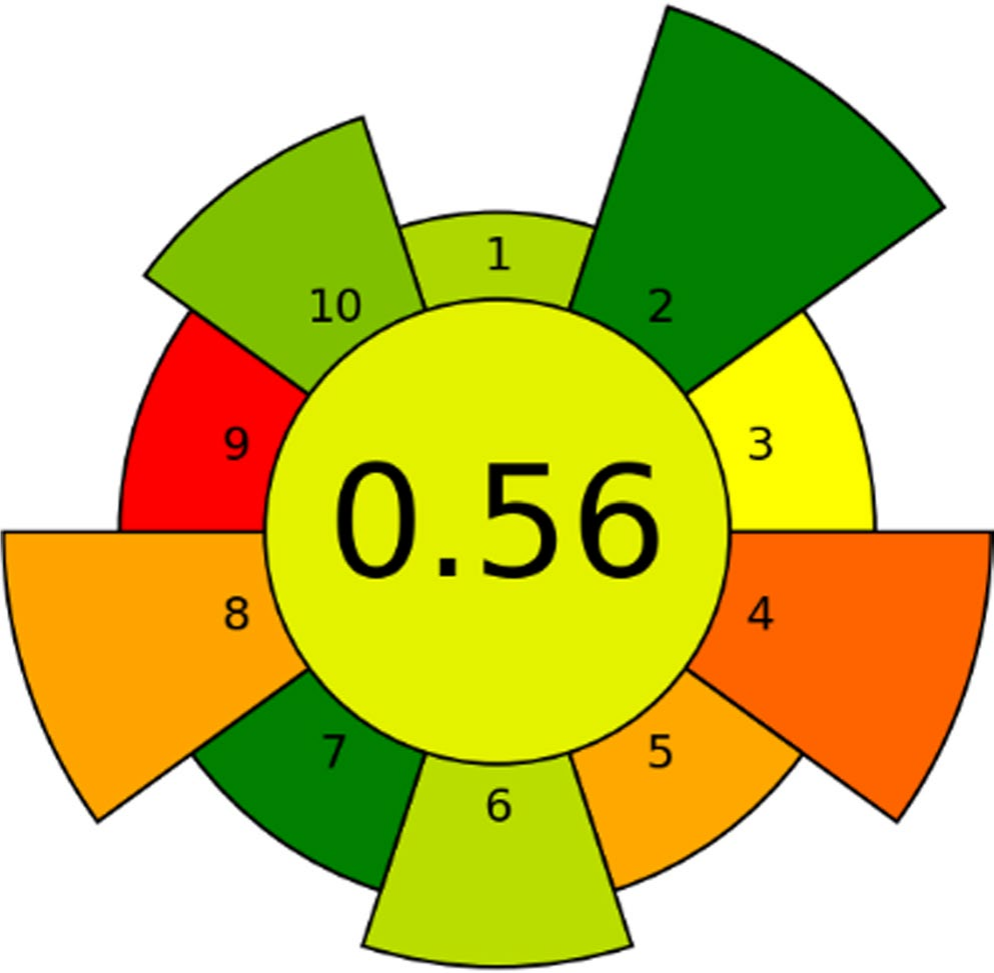
Radial greenness assessment of the proposed analytical procedure based on ten sustainability criteria. Each sector represents a specific parameter of the analytical workflow, the color scale from green to red reflects the degree of environmental friendliness. The central value corresponds to the overall greenness score (0–1 scale). The relatively lower scores observed in some sectors are mainly associated with the energy requirements of the instrumental analysis stage

The method achieved an overall greenness score of 0.56 (Fig. 11) on a scale ranging from 0 to 1, indicating a moderate level of environmental sustainability. Several criteria display favourable scores, reflecting efficient sample preparation steps and relatively limited consumption of reagents and materials. These aspects contribute positively to the overall environmental profile of the methodology. However, some sectors present lower scores (orange and red), which are mainly associated with the instrumental analysis stage. The analytical technique employed relies on a high-performance spectrometric instrument that inherently requires significant energy input for plasma generation and operation of auxiliary components such as vacuum pumps and cooling systems. Consequently, the energy demand of the measurement stage represents one of the main contributors to the environmental footprint of the method.

This limitation is largely intrinsic to the nature of the analytical technique and is commonly observed in advanced elemental analysis platforms. Despite this, the relatively short analysis time per sample and the possibility of high-throughput operation partially mitigate the environmental impact associated with instrumental energy consumption.

Overall, the obtained greenness score indicates that the proposed methodology achieves a reasonable compromise between analytical performance, sensitivity, and environmental sustainability, though further improvements could focus on reducing the energy demand of the instrumental stage or optimizing batch analysis to maximize sample throughput.

## Conclusions

A novel magnetic nanosorbent (M@GONIO) was successfully designed, synthesized, and fully characterized, and subsequently applied as the solid phase in a new automated MSPE-FI-HG-GLS-ICP-MS method for the determination of As and Sb in environmental waters. The proposed online approach, which combines magnetic solid-phase extraction with hydride generation, proved to be highly effective for matrix removal, enabling the reliable analysis of complex samples without the need for salinity adjustment or collision/reaction cell technology. The method was validated using certified reference materials and further applied to water samples of different origins, yielding results in good agreement with literature values.

Experimental conditions were systematically optimized to maximize analytical sensitivity, resulting in a fully automated, simple, rapid, cost-effective, and environmentally friendly methodology. The method provides excellent analytical performance in terms of detection limits, enrichment factors, precision, and sensitivity, particularly for Sb, positioning it among the most competitive approaches reported to date. In addition, low reagent consumption, a reduced sample volume (10 mL), and a high sample throughput (14.4 h⁻¹) make the proposed method especially suitable for routine environmental monitoring. To the best of our knowledge, this is the first report on the synthesis and application of a magnetic nanosorbent specifically developed for the control of As and Sb pollution in environmental waters.

Furthermore, the integration of MSPE with an online HG-ICP-MS configuration minimizes manual handling steps, significantly reducing the risk of contamination and analyte loss, improving overall reproducibility and robustness of the analytical workflow.

The excellent reusability of the nanosorbent, exceeding 200 extraction cycles without significant loss of performance, further highlights its suitability for long-term application in routine monitoring systems and reduces overall operational costs. In addition, the method demonstrates strong environmental compatibility in terms of Green Analytical Chemistry principles, as confirmed by the AGREE assessment, mainly due to the low solvent consumption and high degree of automation.

From an environmental perspective, the method is particularly relevant for areas affected by mining and industrial activities, where arsenic and antimony contamination often coexists at trace and ultratrace levels. Therefore, the proposed approach provides not only an analytical advancement but also a practical tool for environmental surveillance and risk assessment in real-world scenarios.

## Data Availability

The datasets generated and/or analyzed during the current study are available from the corresponding author on reasonable request. All relevant data supporting the findings of this study are included in the article.
